# Health-Related Quality of Life Sleep Score Predicts Transfer to Hemodialysis among Patients on Peritoneal Dialysis

**DOI:** 10.3390/healthcare10061030

**Published:** 2022-06-01

**Authors:** Tomoki Nagasaka, Naoki Washida, Kiyotaka Uchiyama, Eriko Yoshida Hama, Ei Kusahana, Takashin Nakayama, Itaru Yasuda, Kohkichi Morimoto, Hiroshi Itoh

**Affiliations:** 1 Department of Endocrinology, Metabolism and Nephrology, Keio University School of Medicine, 35 Shinanomachi, Shinjuku, Tokyo 160-8582, Japan; n.tomoki0224@gmail.com (T.N.); washida@iuhw.ac.jp (N.W.); erkysd@gmail.com (E.Y.H.); shoot.8.9387@gmail.com (E.K.); takashin.nakayama@gmail.com (T.N.); le5chat7sportif@gmail.com (I.Y.); hiito@keio.jp (H.I.); 2 Department of Nephrology, International University of Health and Welfare Narita Hospital, 4–3 Kozunomori, Narita 286-8686, Japan; 3 Apheresis and Dialysis Center, Keio University School of Medicine, 35 Shinanomachi, Shinjuku, Tokyo 160-8582, Japan; kohkichi.morimoto@keio.jp

**Keywords:** peritoneal dialysis, sleep, health-related quality of life, kidney disease quality of life-short form, transfer to hemodialysis

## Abstract

Despite the superiority of peritoneal dialysis (PD) over hemodialysis (HD) regarding health-related quality of life (HRQOL), the specific HRQOL domain(s) that predict unplanned HD transfer remains uncertain. In this cohort study, we assessed the HRQOL of 50 outpatients undergoing PD using the Japanese version 1.3 Kidney Disease Quality of Life-Short Form from March 2017 to March 2018 and prospectively analyzed the association of each HRQOL component with HD transfer until June 2021. During the follow-up (41.5 (13.0–50.1) months), 21 patients were transferred to HD. In a multivariate Cox proportional hazards model adjusted for age, sex, PD vintage, urine output, Charlson comorbidity index, and incremental shuttle walking test, a higher sleep score was significantly associated with lower HD transfer rates (HR 0.70 per 10, *p* = 0.01). An adjusted subdistribution hazard model where elected transition to HD, death, and transplantation were considered competing events of unintended HD transfer that showed sleep score as an exclusive predictor of HD transfer (HR 0.70 per 10, *p* = 0.002). Our results suggest that sleep score among the HRQOL subscales is instrumental in predicting HD transfer in patients undergoing PD.

## 1. Introduction

In contrast to hemodialysis (HD), in which patients primarily go to the clinic thrice weekly, peritoneal dialysis (PD) can be performed at home, requiring only one or two visits to the hospital per month. Additionally, these PD characteristics allow the treatment to be tailored to the patient’s lifestyle pattern, leading to more time for family, work, and social activities, which greatly improves health-related quality of life (HRQOL). Specifically, a recent meta-analysis reported that patients who use PD had better HRQOL scores compared to patients who use HD, assessed with a Short-Form Health Survey (SF-36), EuroQoL-5-dimension, and Kidney Disease Quality of Life Instrument (KDQOL) [1].

A previous trial called Adequacy of Peritoneal Dialysis in Mexico (ADMEX) demonstrated that the physical (PCS) and mental (MCS) component summary shortened from SF-36 results (a general instrument), including the kidney disease component summary (KDCS) computed from domains of the KDQOL questionnaire (a kidney disease-specific instrument), were highly predictive of survival and hospitalizations among patients undergoing PD [2]. Additionally, a recent Brazilian cohort suggested that the increase in some domains of SF-36, including bodily pain and MCS, was associated with decreased risk of death [3]^.^ However, despite these results that better HRQOL was associated with better outcomes, only a few studies on specific HRQOL components that predict prognosis exists, especially regarding the KDQOL questionnaire.

We previously conducted a cross-sectional study to assess the association between HRQOL and physical capacity, including the distance in the incremental shuttle walking test (ISWT). Then, we clarified the strong positive correlation between various HRQOL domains assessed using the Japanese version 1.3 Kidney Disease Quality of Life-Short Form (KDQOL-SF) and ISWT [4]. However, this cohort study was conducted prospectively using the same population to determine the contribution of each HRQOL component to PD-related outcomes, including time until HD transfer in patients undergoing treatment PD.

## 2. Materials and Methods

### 2.1. Study Population

The Ethics Committee of our hospital (approved number: 20160201/20211113) reviewed and approved the study and all its protocols. Moreover, written informed consent was obtained from all of the patients before participation. Subsequently, we recruited all stable patients aged 20–90 years who received PD for 3 months or more in our hospital between March 2017 and March 2018 for this single-center cohort study. Since the participants were required to undergo exercise testing at baseline, patients considered unsuitable for exercise testing were excluded based on the American College of Sports Medicine and Japanese Circulation Society guidelines, as previously described [4,5,6].

### 2.2. Data Collection, Participant Evaluation, and Biochemical Analyses

Demographic data, including age, sex, PD vintage (years), diabetes mellitus (DM), and the Charlson comorbidity index (CCI), were collected and calculated from medical records during enrollment, whereas exercise parameters, including the total distance of ISWT, body mass index (kg/m^2^), and skeletal mass index (kg/m^2^) using whole-body dual-energy X-ray absorptiometry (DXA; QDR 4500/A, Hologic, Waltham, MA, USA) were measured as previously described [4,7]. Additionally, biochemical data at the time of enrollment, including serum albumin (mg/dL) with geriatric nutritional risk index, intact parathyroid hormone (PTH) (pg/mL), and urea kinetics (Kt/V), were obtained and determined using blood samples and 24-h urine and PD-fluid collections [4,7].

### 2.3. Assessment of Health-Related Quality of Life

HRQOL was assessed at baseline using the Japanese version 1.3 KDQOL-SF, which has undergone translation, cultural adaptation, and initial reliability and multitrait testing of the original KDQOL-SF (US-English) version for use in Japan [8]. KDQOL-SF comprises 79 items: 36 items on SF-36 (general HRQOL) and 43 items on KDQOL (specific to kidney disease and dialysis). SF-36 comprised eight components: physical functioning, physical role functioning, bodily pain, general health, vitality, social functioning, emotional role functioning, and mental health on a 0–100 point score (the higher the score, the better the patient’s HRQOL). Subsequently, the results from the SF-36 instrument were summarized into PCS, MCS, and role/social component summary (RCS) [9]. Then, the mean for each summary scale was set at 50 points in the general population, with a standard deviation of 10 points. However, KDQOL measured 11 domains, also on a 0–100 point score: symptoms/problems, effects of kidney disease, the burden of kidney disease, work status, cognitive function, the quality of social interaction, sexual function, sleep, social support, dialysis staff encouragement, and patient satisfaction. Finally, the KDCS score, also on a 100-point score, was calculated following Mapes et al. [10].

### 2.4. Follow-Up

All of the participants were prospectively followed until the cessation of PD, death, or the end of the study (June 2021). The reasons for the cessation of PD and causes of peritonitis were recorded, along with the relevant dates. Additionally, the primary end-point for the included patients was the transition to HD, which was determined jointly by the patients and physicians due to acute medical problems associated or not associated with PD. Patients who underwent planned HD transfer (or elective modality change) were censored. Moreover, participants who died or underwent kidney transplantation were censored, as previously recommended by the International Society for Peritoneal Dialysis [11].

### 2.5. Statistical Analyses

Continuous variables were expressed as mean ± standard deviation or median (25th–75th percentile) according to normality assessed using the Shapiro–Wilk test and binary variables as frequencies and percentages. Then, we used the adjusted Cox proportional hazard model to evaluate the association of each HRQOL (KDQOL/SF-36) component with time until HD transfer; the results are reported as hazard ratio (HR) and 95% confidence interval. Additionally, we used the subdistribution hazard model proposed by Fine and Gray and the cause-specific hazard model for standard Cox regression as a sensitivity analysis, where elected transition to HD, death, and transplantation were considered competing events of unintended HD transfer [12]. These multivariate analyses were performed with covariates that had previously been reported to be associated with time until HD transfer. In addition to age and sex, DM, CCI, PD vintage, urine output, and ISWT were included as candidate covariates. However, due to the strong and significant correlations observed between DM and CCI, DM and CCI were included separately, taking multicollinearity into account: CCI was included in model A, whereas DM was included in model B [12,13,14,15]. Furthermore, we added the prespecified Pearson’s correlation analysis to assess the correlation between HRQOL scores, which were independently associated with an unplanned transition to HD, and continuous clinical parameters. Additionally, the HRQOL scores were compared between binary clinical variables, including sex and PD modality.

## 3. Results

### 3.1. Clinical Characteristics

**Figure 1:**
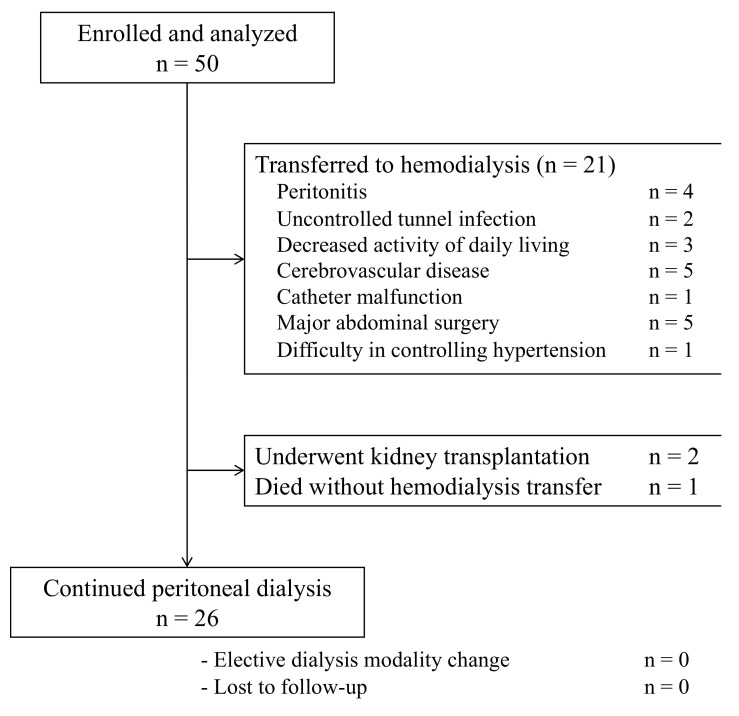
Flow chart of the study participants.

Among 50 patients who consented to participate in the study and enrolled, [4,7] 21 (42%) patients discontinued PD and were transferred to HD during the study period. The causes of transfer were peritonitis (n = 10), uncontrolled tunnel infection (n = 2), difficulties performing PD due to decreased activities of daily living (n = 3) or cerebrovascular disease leading to physical disability (n = 3), catheter malfunction (n = 1), major abdominal surgery (n = 1), and difficulty in controlling hypertension with volume overload (n = 1) (Figure 1). Furthermore, while one participant underwent kidney transplantation, two died without changing PD modality. The results also showed that no patient underwent a transition to HD in a planned fashion. The median follow-up period was 41.5 (13.0–50.1) months, and no loss to follow-up was experienced.

Baseline clinical characteristics and HRQOL (KDQOL/SF-36) components are summarized in Table 1 and Table 2, respectively. Briefly, the median age of patients in our cohort was 63 years, and they were predominantly male (74%). The participants comprised 13 (26%) patients treated with automated PD (APD) and 37 (74%) patients treated with continuous ambulatory PD (CAPD). Moreover, HRQOL domains with lower scores included burden of kidney disease, sleep, general health, vitality, and PCS (43.3 ± 21.5, 62.2 ± 18.2, 44.5 ± 17.7, 54.8 ± 20.8, and 39.8 ± 13.8, respectively).

### 3.2. Association of HRQOL (KDQOL/SF-36) Components with HD Transfer

Among KDQOL and SF-36 domains in the fully adjusted multivariate Models A and B using the Cox proportional hazards model, only the sleep score showed a significant association with HD transfer (HR 0.69 [per 10], *p* = 0.01 and HR 0.72 [per 10], *p* = 0.02, respectively) (Table 3). These models assumed a proportional hazard assumption and had a sufficiently high concordance index of approximately 0.80.

The results of sensitivity analysis using the subdistribution hazard model were also similar to those of the Cox proportional hazards model analyses. In Models A and B, only the sleep score was significantly associated with HD transfer (HR 0.70 [per 10], *p* = 0.002 and HR 0.73 [per 10], *p* = 0.004, respectively) (Table 3).

### 3.3. Association of Clinical Parameters with HRQOL Sleep Score

There were no significant differences in sleep score between male and female, continuous ambulatory PD (CAPD) and automated PD (APD), or with and without DM (60.0 ± 18.4 vs. 68.3 ± 16.8, *p* = 0.16; 61.7 ± 17.8 vs. 63.5 ± 20.2, *p* = 0.77; 64.4 ± 16.4 vs. 57.1 ± 21.7, *p* = 0.20, respectively) nor between the other binary variables. Among the continuous clinical parameters, only serum potassium had a significant negative correlation with the sleep score (r = −0.34, *p* = 0.01), whereas serum phosphorus and PTH had inverse associations with borderline significance with sleep score (r = −0.22, *p* = 0.13 and r = −0.26, *p* = 0.07, respectively).

## 4. Discussion

This prospective cohort study demonstrated that symptoms/problems and sleep among KDQOL domains and vitality among SF-36 domains were significantly associated with HD transfer in univariate analysis. However, only the sleep score has remained a significant predictor of PD withdrawal in a fully adjusted multivariate model among these domains. Specifically, an increase in the sleep score was associated with an increase in time until HD transfer. Although the ADEMEX trial [2] and a Brazilian cohort study [3] suggested an association between several HRQOL domains and mortality or hospitalizations, this study was the first to focus on the associations between specific HRQOL domains, including KDQOL components and HD transfer in Japanese patients undergoing PD.

A recent meta-analysis reported that patients undergoing PD had better HRQOL assessments with SF-36 and KDQOL than patients on hemodialysis, especially regarding the components including MCS, symptoms/problems, and burden of kidney disease [1]. Moreover, a Korean nationwide cohort study suggested that although patients on both HD and PD experienced significant decreases in the HRQOL domains over two years after the initiation of dialysis, several HRQOL domains assessed with KDQOL-SF were still higher in patients on PD than those on HD at one and two years [16]. Additionally, even in older patients with whom the default dialysis modality was commonly HD, no difference in HRQOL measures was observed between those treated with assisted PD and those treated with HD [17]. Furthermore, treatment satisfaction was even higher in patients undergoing PD. Hence, we consider that these advantages of PD over HD in HRQOL can be attributed to PD being performed at home, requiring fewer visits to the hospital, and allowing treatment to be tailored to the patient’s lifestyle. A recent guideline suggested that the goal of caring for patients undergoing PD should be directed at adjusting therapy to maximize the patient’s HRQOL and incorporate the routine assessments of HRQOL into the care of these patients as an important tool for assessing the adequacy of dialysis markers [18]. However, other systemic reviews comparing HRQOL between PD and HD suggested a high heterogeneity among studies, and there was inconclusive evidence of the superiority of PD over HD with respect to the HRQOL [19,20]. Although a randomized controlled trial is necessary to provide definitive evidence in this respect, random assignment of patients to PD or HD is fraught with ethical concerns, and no such trial has been conducted, nor will it be conducted in the future.

Alternatively, the predictors of HRQOL and how to improve HRQOL in patients treated with PD have not been fully elucidated [21,22]. Overall, the sex of the patient affects the HRQOL subscales. Most recently, the Dialysis Outcomes and Practice Patterns Study (DOPPS) and the Peritoneal Dialysis Outcomes and Practice Patterns Study reported that the female sex was associated with lower PCS and a higher burden of kidney disease score [23]. However, in this study, we observed no significant difference between male and female patients with respect to sleep scores. In a previous study, we demonstrated a strong relationship between aerobic capacity assessed with ISWT and HRQOL in patients with PD [4]. A similar cross-sectional study from China also reported that measuring physical performance using the timed up and go test was significantly associated with mental HRQOL [24]. However, our randomized controlled trial showed that home-based exercise significantly improved several HRQOL domains assessed with KDQOL-SF, including KDCS, physical role functioning, emotional role functioning, and RCS, but not the sleep score [25]. Furthermore, the impact of PD modality (CAPD/APD) or dialysis adequacy on HRQOL remains controversial, including results from the ADMEX trial [2,22].

Specifically, regarding sleep, the prevalence of sleep disturbances was higher in patients undergoing PD than in healthy controls [26]. Sleep disorders in patients on PD include altered sleep architecture, sleep apnea, restless legs syndrome, periodic limb movement disorder, and excessive daytime sleepiness [27]. In elderly patients undergoing PD, higher hemoglobin and lower serum phosphorus were significantly correlated with better sleep efficiency, and age was the only significant predictor of the total sleep time [28]. Furthermore, although APD is more likely to interrupt sleep compared with CAPD, it has been considered effective for sleep apnea [29]. In our study, the sleep score was not significantly different between patients undergoing CAPD and APD, and only high serum potassium was a significant predictor of worse sleep scores, which has also been suggested by a previous study on HD patients in whom serum potassium of over 5.00 mEq/L was found to contribute to sleep disturbances [30].

Several studies have investigated the association between sleep quality and outcomes among patients undergoing dialysis. Data from a national prospective cohort in U.S. indicated that a decline in sleep quality during the first year on dialysis, and not baseline sleep quality, was associated with worse survival in patients on either HD or PD [31]. In a small-scale study conducted in China on both HD and PD patients, worse sleep quality, as assessed with the Pittsburgh Sleep Quality Index score, showed an independent association with the increased risk of all-cause mortality [32]. The association of sleep quality with outcomes is likely to be observed among exclusively PD patients; however, there is a paucity of data in this respect. In a recent study, among the symptoms (including anorexia, insomnia, fatigue, and nausea) experienced by patients undergoing PD, only nausea showed a significant association with all-cause mortality despite a high prevalence of insomnia (32.7%) in PD patients [33]. Then, we also investigated for the first time the association of HRQOL sleep score with HD transfer, which is an important outcome for PD patients in addition to death. We found that the sleep score was a predictor of HD transfer. Further studies are required to provide more definitive evidence of the prognostic value of sleep for outcomes, including all-cause mortality. Additionally, although improving sleep quality may improve outcomes in PD patients, the evidence for improving sleep quality for patients with CKD is sparse [34]. Therefore, further trials are necessary to elucidate effective interventions to improve HRQOL, particularly the sleep component, in patients undergoing PD.

This study has several limitations. First, this was an observational cohort study, which makes it difficult to prove the causality between sleep scores and outcomes in patients undergoing PD. Second, this was a single-center study with a relatively small sample size. This limited sample size may render the results less inconclusive; however, a single-center study may have the merit of less confounding factors with respect to patient-reported outcome measures, including HRQOL. Moreover, the sufficiently high concordance values in the multivariate analyses suggested that the model was stable. Third, our criteria for excluding patients unsuitable for exercise testing may partly decrease the generalizability of our findings. Additionally, no patients were excluded from the study because of their age. Thus, the actual age range in our cohort was quite wide (38–80 years), which may have affected our results. However, we performed multivariate analyses adjusted for various parameters, including age, which may have helped minimize the effect of age on the results. The effect of sex, which may affect the outcome, was also minimized similarly. Finally, the sleep score among KDQOL domains itself could not assess the character of sleep disturbances, including decreased sleep duration, quality, or apnea, unlike polysomnography and other sleep-specific questionnaires [35].

## 5. Conclusions

In conclusion, we demonstrated that an increase in the sleep score among the HRQOL subscales significantly and independently predicted a reduced risk of HD transfer in patients undergoing maintenance PD. However, our results should be generalized cautiously due to many limitations, possible selection bias, and subsequent statistical analysis. Additionally, further studies should verify what interventions improve the sleep of patients undergoing PD and whether those interventions can reduce PD withdrawal.

## Figures and Tables

**Table 1:** Baseline clinical characteristics of the study participants.

Variables	Total (n = 50)	Continued PD (n = 29)	Transferred to HD (n = 21)
Age, year	63 (59–71)	63 (58–70)	63 (60–70)
Sex (% male)	37 (74%)	21 (81%)	16 (76%)
DM	15 (30%)	5 (19%)	10 (48%)
CCI	3.0 (2.0–4.0)	2.5 (2.0–3.0)	3.7 (3.0–5.0)
APD/CAPD	13/37 (26/74%)	8/18 (31/69%)	5/16 (24/76%)
Cardiovascular disease	16 (32%)	4 (15%)	12 (57%)
Smoking history	28 (56%)	15 (58%)	13 (62%)
PD vintage, year	3.5 (1.3–6.5)	3.7 (1.2–5.8)	4.0 (1.5–6.6)
Urine output, mL/day	410 (0–1,050)	732 (7–1,150)	485 (0–900)
Renal, Kt/V	0.19 (0–0.80)	0.43 (0–0.84)	0.39 (0–0.73)
BMI, kg/m^2^	23.8 ± 4.0	23.5 ± 4.0	24.2 ± 4.1
SMI, kg/m^2^	7.3 ± 1.3	7.4 ± 1.2	7.1 ± 1.3
PD, Kt/V	1.29 (1.01–1.57)	1.27 (0.97–1.55)	1.39 (1.07–1.58)
Total, Kt/V	1.70 (1.44–1.89)	1.70 (1.47–1.87)	1.76 (1.43–1.91)
Mean blood pressure, mmHg	96.9 ± 13.8	97.3 ± 13.0	96.4 ± 15.2
Hemoglobin, g/dL	10.6 ± 1.3	10.5 ± 1.3	10.8 ± 1.2
Albumin, mg/dL	3.4 ± 0.5	3.4 ± 0.5	3.4 ± 0.4
Potassium, mEq/L	4.6 ± 0.6	4.6 ± 0.6	4.6 ± 0.7
Calcium, mg/dL	9.6 ± 1.6	9.7 ± 2.0	9.5 ± 0.6
Phosphorus, mg/dL	5.5 ± 1.1	5.2 ± 1.2	5.8 ± 1.0
PTH, pmol/L	184.0 (106.3–270.8)	224.1 (101–271)	216.1 (110–270)
CRP, mg/L	0.09 (0.03–0.23)	0.24 (0.02–0.22)	0.28 (0.04–0.23)
hANP, pg/mL	70.4 (49.1–118.8)	87.7 (41.6–120)	117.8 (50.2–115)
ISWT, m	312.0 ± 138.2	353.2 ± 132.7	255.2 ± 124.0
Handgrip strength, kg	28.5 (23.3–31.0)	28.1 (25.9–31.7)	26.6 (21.6–30.7)
Quadriceps strength, kg	22.3 (14.5–31.1)	24.8 (15.7–34.6)	21.2 (14.5–27.7)

PD = peritoneal dialysis; HD = hemodialysis; DM = diabetes mellitus; CCI = Charlson comorbidity index; APD = automated peritoneal dialysis; CAPD = continuous ambulatory peritoneal dialysis; BMI = body mass index; SMI = skeletal mass index; PTH = parathyroid hormone; CRP = C-reactive protein; hANP = human atrial natriuretic peptide; ISWT = incremental shuttle walking test.

**Table 2:** Health-related quality of life (KDQOL/SF-36) scores of the study participants.

HRQOL Components	Total (n = 50)	Continued PD (n = 29)	Transferred to HD (n = 21)
KDQOL			
Symptoms/problems	77.6 ± 12.5	80.9 ± 11.4	73.0 ± 12.8
Effects of kidney disease	76.7 ± 14.4	80.0 ± 14.3	72.1 ± 13.6
Burden of kidney disease	43.3 ± 21.5	47.4 ± 23.5	37.5 ± 17.2
Cognitive function	90.4 ± 12.2	92.4 ± 12.8	87.5 ± 11.0
Quality of social interaction	87.8 ± 15.8	89.4 ± 14.5	85.6 ± 17.8
Sleep	62.2 ± 18.2	67.2 ± 17.5	55.3 ± 17.3
Social support	80.0 ± 20.2	79.3 ± 21.2	80.9 ± 19.2
Encouragement from staff	82.8 ± 19.3	86.0 ± 15.9	78.4 ± 23.0
Satisfaction for care	80.6 ± 16.2	82.7 ± 17.3	77.5 ± 14.6
KDCS	74.1 ± 9.9	76.4 ± 9.8	71.0 ± 9.2
SF-36			
Physical function	73.7 ± 21.5	74.5 ± 23.2	72.6 ± 19.3
Physical Role Functioning	68.5 ± 24.0	72.8 ± 23.4	62.5 ± 24.0
Pain	71.1 ± 24.3	73.3 ± 25.7	68.1 ± 22.4
General health	44.5 ± 17.7	46.3 ± 19.4	42.1 ± 15.2
Vitality	54.8 ± 20.8	59.5 ± 19.3	48.5 ± 21.6
Social Functioning	69.0 ± 28.0	67.7 ± 31.3	70.8 ± 23.5
Emotional Role Functioning	77.5 ± 21.6	81.0 ± 20.0	72.6 ± 23.4
Mental health	71.3 ± 18.1	74.9 ± 18.0	66.4 ± 17.6
PCS	39.8 ± 13.8	40.5 ± 15.0	38.8 ± 12.3
MCS	50.0 ± 8.9	51.4 ± 8.3	48.2 ± 9.6
RCS	44.3 ± 12.4	45.2 ± 12.0	43.1 ± 13.2

PD = peritoneal dialysis; HD = hemodialysis; HRQOL = health-related quality of life; KDQOL = kidney disease quality of life; SF-36 = 36-item Short-Form Health Survey medical outcomes study; KDCS = kidney disease component summary; PCS = physical component summary; MCS = mental component summary; RCS = role/social component summary.

**Table 3:** Association of health-related quality of life (KDQOL/SF-36) components with hemodialysis transfer using adjusted standard Cox regression models and Fine and Gray subdistribution hazard models (models A and B).

HRQOL Domains (per 10)	Model A			Model B		
	HR	95% CI	*p*-Value	HR	95% CI	*p*-Value
Cause-specific hazards						
KDQOL						
Symptoms/problems	0.82	0.53–1.28	0.39	0.72	0.47–1.11	0.14
Effects of kidney disease	1.08	0.77–1.52	0.65	1.00	0.71–1.40	0.99
Burden of kidney disease	1.11	0.79–1.54	0.56	0.97	0.73–1.28	0.81
Cognitive function	1.03	0.68–1.55	0.91	0.91	0.62–1.35	0.65
Quality of social interaction	0.96	0.72–1.29	0.80	0.97	0.72–1.30	0.81
Sleep	0.69	0.52–0.92	0.01	0.72	0.55–0.94	0.02
Social support	1.20	0.92–1.56	0.18	1.16	0.89–1.49	0.27
Encouragement from staff	0.87	0.66–1.14	0.30	0.87	0.65–1.17	0.36
Satisfaction for care	0.78	0.54–1.12	0.18	0.87	0.63–1.21	0.41
KDCS	0.90	0.46–1.75	0.76	0.93	0.52–1.66	0.81
SF-36						
Physical function	1.22	0.97–1.54	0.09	1.16	0.92–1.45	0.21
Physical Role Functioning	0.91	0.69–1.20	0.50	0.91	0.70–1.18	0.48
Pain	0.98	0.79–1.20	0.81	0.96	0.79–1.18	0.71
General health	1.36	0.92–2.02	0.12	1.13	0.81–1.58	0.46
Vitality	0.89	0.64–1.22	0.47	0.86	0.64–1.14	0.29
Social Functioning	1.08	0.89–1.31	0.45	1.06	0.89–1.27	0.51
Emotional Role Functioning	0.82	0.63–1.07	0.14	0.85	0.67–1.09	0.20
Mental health	0.84	0.64–1.10	0.21	0.89	0.70–1.13	0.34
PCS	1.35	0.95–1.93	0.10	1.23	0.86–1.76	0.25
MCS	0.81	0.45–1.46	0.49	0.79	0.45–1.38	0.41
RCS	0.71	0.43–1.16	0.17	0.83	0.54–1.28	0.40
Subdistribution hazards						
KDQOL						
Symptoms/problems	0.84	0.52–1.35	0.47	0.76	0.48–1.18	0.22
Effects of kidney disease	1.12	0.81–1.53	0.50	1.04	0.75–1.43	0.83
Burden of kidney disease	1.14	0.76–1.72	0.53	1.00	0.75–1.34	1
Cognitive function	1.02	0.67–1.57	0.92	0.90	0.60–1.34	0.61
Quality of social interaction	0.97	0.70–1.34	0.85	0.97	0.71–1.32	0.84
Sleep	0.70	0.55–0.88	0.002	0.73	0.60–0.90	0.004
Social support	1.17	0.94–1.46	0.17	1.13	0.87–1.47	0.37
Encouragement from staff	0.89	0.73–1.09	0.25	0.90	0.68–1.19	0.46
Satisfaction for care	0.81	0.63–1.04	0.10	0.91	0.71–1.18	0.48
KDCS	0.96	0.46–2.01	0.91	1.00	0.59–1.70	1
SF-36						
Physical function	1.20	0.98–1.48	0.07	1.14	0.93–1.41	0.22
Physical Role Functioning	0.92	0.65–1.31	0.65	0.92	0.69–1.25	0.61
Pain	0.98	0.78–1.24	0.88	0.97	0.70–1.20	0.80
General health	1.33	0.89–1.98	0.17	1.13	0.81–1.58	0.47
Vitality	0.91	0.62–1.34	0.63	0.87	0.63–1.21	0.41
Social Functioning	1.09	0.89–1.32	0.42	1.07	0.91–1.26	0.41
Emotional Role Functioning	0.84	0.61–1.14	0.26	0.88	0.69–1.12	0.29
Mental health	0.83	0.63–1.09	0.18	0.88	0.70–1.10	0.26
PCS	1.34	1.00–1.78	0.05	1.21	0.87–1.70	0.26
MCS	0.82	0.45–1.49	0.51	0.79	0.45–1.40	0.42
RCS	0.72	0.39–1.36	0.32	0.86	0.53–1.38	0.53

Model A was adjusted for age, sex, PD vintage, urine output, CCI, and ISWT. Of these variables, DM replaced CCI in adjusted Model B. HRQOL = health-related quality of life; KDQOL = kidney disease quality of life; HR = hazard ratio; CI = confidence interval; SF-36 = 36-item Short-Form Health Survey medical outcomes study; KDCS = kidney disease component summary; PCS = physical component summary; MCS = mental component summary; RCS = role/social component summary; DM = diabetes mellitus; CCI = Charlson comorbidity index; ISWT = incremental shuttle walking test.

## Data Availability

Original data are available from the corresponding author on reasonable request.
